# Pathogen-specific clinical and inflammatory phenotypes in pediatric catheter-related infections

**DOI:** 10.1007/s00431-026-07098-9

**Published:** 2026-05-28

**Authors:** Merve Özden Budağ, Tugce Tural-Kara

**Affiliations:** 1 Department of Pediatrics, Akdeniz University Hospital, Antalya, Turkey; 2 Department of Pediatric Infectious Disease, Akdeniz University Hospital, Antalya, Turkey

**Keywords:** Catheter-related infections, Pediatrics, Inflammatory biomarkers, Pathogen stratification, Risk factors

## Abstract

Early empirical management of pediatric catheter-related infections (CRI) is complicated by delayed microbiological confirmation and heterogeneous local pathogen epidemiology. Pathogen-specific clinical and inflammatory profiling may facilitate contextual risk assessment at the time of clinical suspicion. In this retrospective single-center cohort study, 291 CRI episodes contributed by 177 pediatric patients between March 1, 2020, and February 29, 2024, were analyzed. Clinical variables at catheter insertion and inflammatory biomarkers at infection onset were evaluated. CRI episodes were classified according to the first clinically significant isolate. Multivariable logistic regression models explored predictors of (i) Gram-negative versus Gram-positive CRI and (ii) fungal versus bacterial CRI. During 24,846 catheter days of follow-up, the incidence density (rate per 1000 catheter days) was 11.7 per 1000 catheter days (95% CI 10.4–13.1). A causative pathogen was identified in 94.2% of episodes: 51.1% Gram-positive, 40.5% Gram-negative, and 8.4% fungal. Gram-negative infections were associated with higher CRP, PCT, and NLR levels. PCT demonstrated the highest discriminatory capacity for Gram-negative CRI (AUC 0.663, 95% CI 0.593–0.734). Lymphocyte count showed moderate discrimination for fungal CRI (AUC 0.711).

*Conclusions*: Pediatric CRI exhibit pathogen-associated inflammatory and device-related patterns; however, discriminatory performance of individual biomarkers remains modest. These findings may support contextual risk assessment at first suspicion but do not justify modification of guideline-based empirical therapy without prospective validation.

**Table:** 

**What is Known:** •*Pediatric catheter-related infections (CRI) are prevalent in tertiary care settings, particularly among children with hematologic or oncologic disorders.* •*The early clinical presentation of the condition is often non-specific, and microbiological confirmation is frequently delayed, necessitating empirical therapy.* •*Gram-positive organisms predominate; however, Gram-negative and fungal pathogens are increasingly reported.* •*Inflammatory biomarkers, including CRP and procalcitonin, are frequently utilized in clinical practice, though their capacity to differentiate among different pathogen classes remains equivocal.* **What is New:** •*In a cohort of 177 children, contributing 291 CRI episodes, the incidence density was 11.7 per 1000 catheter days.* •*Distinct pathogen-associated phenotypes were identified: Gram-negative CRI exhibited a more pronounced inflammatory profile, while fungal CRI were more frequently associated with temporary and multi-lumen devices.* •*Despite the presence of statistically significant associations, the capacity for discrimination between pathogen classes based on biomarkers was modest (AUC ≤ 0.66 for Gram-negative differentiation), underscoring the limitations of relying on a solitary biomarker.* •*These findings support the implementation of structured, multivariable contextual assessment as a preferred approach over decision-making based exclusively on biomarkers at the initial stage of clinical suspicion.*

**What is Known:**

•*Pediatric catheter-related infections (CRI) are prevalent in tertiary care settings, particularly among children with hematologic or oncologic disorders.*

•*The early clinical presentation of the condition is often non-specific, and microbiological confirmation is frequently delayed, necessitating empirical therapy.*

•*Gram-positive organisms predominate; however, Gram-negative and fungal pathogens are increasingly reported.*

•*Inflammatory biomarkers, including CRP and procalcitonin, are frequently utilized in clinical practice, though their capacity to differentiate among different pathogen classes remains equivocal.*

**What is New:**

•*In a cohort of 177 children, contributing 291 CRI episodes, the incidence density was 11.7 per 1000 catheter days.*

•*Distinct pathogen-associated phenotypes were identified: Gram-negative CRI exhibited a more pronounced inflammatory profile, while fungal CRI were more frequently associated with temporary and multi-lumen devices.*

•*Despite the presence of statistically significant associations, the capacity for discrimination between pathogen classes based on biomarkers was modest (AUC ≤ 0.66 for Gram-negative differentiation), underscoring the limitations of relying on a solitary biomarker.*

•*These findings support the implementation of structured, multivariable contextual assessment as a preferred approach over decision-making based exclusively on biomarkers at the initial stage of clinical suspicion.*

## Introduction

Central venous catheters (CVCs) are integral to modern pediatric care, providing reliable vascular access for chemotherapy, parenteral nutrition, vasoactive therapy, extracorporeal support, and invasive monitoring [1–3]. Their widespread use—particularly in pediatric oncology and critical care—has enabled delivery of life-sustaining therapies and improved continuity of care [4]. However, dependence on long-term intravascular devices has inevitably increased the incidence of catheter-related complications [5].

Among late complications, catheter-related infections (CRI) remain the most clinically consequential, contributing to substantial morbidity, prolonged hospitalization, and mortality [6, 7]. The microbiological spectrum is heterogeneous, encompassing Gram-positive cocci (notably *Staphylococcus aureus* and coagulase-negative *staphylococci*), Gram-negative bacilli (including *Enterobacteriaceae* and non-fermenters), and fungal pathogens, predominantly *Candida* species [8]. Biofilm formation further complicates management by promoting microbial persistence and relapse [9].

Despite this microbiological diversity, early clinical manifestations are largely nonspecific. Fever, hemodynamic instability, and local inflammatory signs do not reliably distinguish pathogen class, and definitive microbiological identification is often delayed [10]. As a result, empirical antimicrobial therapy must be initiated in the setting of diagnostic uncertainty, and catheter management decisions frequently precede pathogen confirmation [11, 12]. In immunocompromised children, delays in adequate therapy may adversely affect outcomes, underscoring the need for early risk stratification [13, 14].

Most pediatric studies have focused on risk factors for CRI occurrence compared with non-infected catheter use [15, 16].

In contrast, relatively few investigations have addressed pathogen-class discrimination among confirmed CRI episodes—a distinction that is directly relevant to empirical treatment strategies [8, 11]. Whether distinct inflammatory and device-associated phenotypes can meaningfully differentiate Gram-negative, Gram-positive, and fungal CRI at the time of clinical suspicion remains insufficiently defined.

The present study aimed to characterize pathogen-class–associated clinical features and inflammatory biomarker profiles in pediatric CRI. We further evaluated the discriminatory performance of these variables using receiver operating characteristic (ROC) analysis and area under the curve (AUC) metrics. We hypothesized that pediatric CRI demonstrate organism-specific inflammatory and device-related patterns, and that integrating these variables into multivariable models might enhance early Gram-negative risk discrimination beyond that achieved by individual biomarkers alone.

## Materials and methods

### Study design and setting

This retrospective observational cohort study included pediatric CRI episodes diagnosed between March 1, 2020, and February 29, 2024, at a tertiary-care academic center. The unit of analysis was individual CRI episodes rather than patients. The study protocol was approved by the institutional ethics committee (November 15, 2023; KAEK-874).

### Case definition and eligibility

CRI was operationally defined according to CDC/NHSN surveillance criteria for central line-associated bloodstream infection (CLABSI), based on laboratory-confirmed bloodstream infection (LCBI) definitions [17]. LCBI required at least one positive blood culture in the absence of an alternative infection source and compatible clinical findings [18]. For common skin commensals, ≥ 2 positive blood cultures from separate samples were required.

Eligible episodes were restricted to cases in which catheter insertion, infection diagnosis, and management occurred within our institution to ensure diagnostic and therapeutic consistency. Peripheral catheter-related infections and externally inserted central venous catheters were excluded.

When multiple CRI episodes occurred in the same patient, episode classification and independence were evaluated in accordance with CDC/NHSN Repeat Infection Timeframe (RIT) criteria. Episodes occurring within the 14-day RIT were considered part of the same infection event and were not counted as separate episodes. Only infections occurring outside the RIT with evidence of clinical and microbiological resolution were considered independent events [19].

In polymicrobial episodes, classification was based on the clinically dominant isolate, determined by microbiological yield, consistency across cultures, and concordance with clinical presentation, with prioritization of fungal isolates when present. Fungal isolates were considered clinically significant when *Candida* spp. were recovered from at least one blood culture in the presence of compatible systemic findings and absence of an alternative infection source, consistent with invasive infection. As *Candida* species are rarely considered blood culture contaminants, their detection was prioritized in pathogen classification. In contrast, isolates identified in a single culture without clinical correlation were interpreted cautiously and evaluated individually based on overall clinical context, host factors, and microbiological data to minimize potential misclassification. Culture-negative episodes were included in descriptive analyses but excluded from pathogen-class regression models. Antimicrobial susceptibility testing was interpreted according to EUCAST clinical breakpoints in use during the study period, with reference to version 13.0 (2023) for standardization [20].

The incidence density (rate per 1000 catheter days) was calculated as the number of CRI episodes per 1000 catheter days, consistent with CDC/NHSN surveillance methodology [10, 21]. Exact Poisson confidence intervals were computed. Neutropenia was defined as an absolute neutrophil count < 1500/mm^3^ (< 1000/mm^3^ in infants < 1 year).

### Data collection and definitions

Data were extracted from institutional electronic medical records. At catheter insertion, recorded variables included demographics, underlying disease, indication for catheterization, insertion site, performing department, catheter type, lumen number, immunosuppression status, and peri-insertion antibiotic exposure. Antibiotic exposure was defined as systemic antibacterial therapy administered within 72 h before or after catheter placement.

At CRI onset (defined as first clinical suspicion with same-day blood sampling), data on CRI subtype, co-infections, inflammatory biomarkers, therapeutic interventions (including catheter removal), and outcomes were collected.

Remission was defined as resolution of clinical signs without recurrence within 30 days [22]. Relapse was defined as recurrent CRI caused by the same organism within 90 days after completion of therapy [23, 24]. Thirty-day mortality was defined as all-cause mortality within 30 days of CRI diagnosis.

Because early catheter removal resulted in complete separation in fungal CRI, catheter removal variables were not entered into the fungal regression model. Given the limited number of fungal events (*n* = 23), the fungal model was constructed parsimoniously.

### Statistical analysis

Analyses were performed using SPSS version 25.0 (IBM Corp., Armonk, NY, USA). Categorical variables are presented as frequencies and percentages and were compared using *χ*^2^ or Fisher’s exact tests as appropriate. Continuous variables were assessed for normality using the Shapiro–Wilk test and are reported as mean ± standard deviation or median (interquartile range). Non-normally distributed variables were compared using Mann–Whitney *U* or Kruskal–Wallis tests.

Multivariable logistic regression models were developed to evaluate predictors of (i) Gram-negative versus Gram-positive CRI and (ii) fungal versus bacterial CRI. Candidate predictors were selected a priori based on biological plausibility and prior literature rather than solely on univariable statistical significance. The primary Gram-negative model included CRP, NLR, lymphocyte count, antibiotic exposure at insertion, immunosuppression status, catheter type, and lumen number. All tests were two-sided, and *p* < 0.05 was considered statistically significant. Missing data were handled using complete-case analysis.

Given the retrospective design and sample size constraints, internal validation (e.g., bootstrapping) was not performed; therefore, potential model optimism cannot be excluded. The limited number of fungal episodes restricted events-per-variable ratios and may increase overfitting risk; estimates should be interpreted cautiously.

Because multiple CRI episodes could occur in the same patient, observations were not strictly independent. However, due to sample size constraints and model stability considerations, clustered or mixed-effects modeling was not applied; this potential intraclass correlation is acknowledged as a limitation.

## Results

### Study population and baseline characteristics

Among 315 screened cases, 24 episodes were excluded as contaminants, yielding 291 evaluable CRI episodes. The mean age at infection was 82.2 ± 59.2 months, and 61.5% of patients were male. Underlying hematologic, oncologic, or immunologic disorders were present in 76.3% of cases, with leukemia or lymphoma accounting for 39.2%.

A total of 177 individual patients contributed 291 CRI episodes. Among these, 112 patients (63.3%) experienced a single episode, whereas 65 patients (36.7%) had two or more episodes during the study period. The mean number of episodes per patient was 1.64. Given this episode-based analytical framework, within-patient clustering may be present and should be considered when interpreting regression estimates.

At the time of catheter insertion, 70.8% of patients were immunosuppressed, most commonly due to corticosteroid therapy (38.1%). Prior surgery was documented in 41.9% of cases, total parenteral nutrition (TPN) in 9.6%, skin integrity disruption in 3.8%, and neutropenia in 37.4%.

During 24,846 catheter days of follow-up, 291 CRI episodes were identified, corresponding to an incidence density of 11.71 per 1000 catheter days (95% CI 10.40–13.14).

### Clinical presentation and timing

Fever was the most common presenting feature (74.1%), followed by erythema or rash (14.4%), respiratory failure (5.9%), altered mental status (5.5%), and hypotension (3.1%). Markers of systemic severity—respiratory failure and altered consciousness—were more frequent in Gram-negative and fungal CRI than in Gram-positive infections (*p* = 0.02).

The median interval from catheter placement to infection onset was 41 days (mean 85.3 ± 108.1; range 1–628). Early-onset CRI (≤ 7 days) occurred in 13.7% of episodes, whereas 86.3% were late-onset. Timing of infection was not associated with pathogen class (*p* = 0.2).

### Catheter characteristics

The most common indications for catheter placement were planned long-term therapy (36.8%), difficult peripheral access (28.9%), and catheter revision (26.5%). Permanent devices accounted for 76.3% of catheters, and 82.1% were double-lumen lines. Hickman catheters were most frequently used (69.1%). Most insertions involved upper-body sites (94.8%), predominantly the jugular vein (83.8%).

CLABSI constituted 90.4% of CRI episodes, with exit-site (6.2%), tunnel (3.1%), and pocket infections (0.3%) comprising the remainder. Device-related characteristics are summarized in Table 1.

**Table 1:** Clinical and catheter-related characteristics in children with catheterrelated infection (CRI)

	***n***	**%**
**Catheterization indication**		
Planned long‑term therapy	107	36.8
Difficult peripheral access	84	28.9
Revision of existing line	77	26.5
Hemodialysis	19	6.5
Intra‑operative placement	4	1.3
**Catheter insertion site**		
Upper-body sites	276	94.8
Lower-body sites	15	5.2
**Vascular site**		
Jugular vein	244	83.8
Subclavian vein	32	11.0
Femoral vein	15	5.2
**Department performing insertion**		
Interventional radiology	206	70.8
Intensive care unit	50	17.2
Pediatric surgery	22	7.6
Anesthesiology	10	3.4
Pediatric wards	3	1.0
**Catheter type**		
Permanent	222	76.3
Temporary	69	23.7
**Catheter function**		
Hickman	201	69.1
Temporary CVC	52	17.9
Hemodialysis	31	10.7
Broviac	7	2.3
**Number of lumens**		
2	239	82.1
3	52	17.9
**Type of CRI**		
Central‑line–associated bloodstream infection	263	90.4
Exit‑site infection	18	6.2
Tunnel infection	9	3.1
Pocket infection	1	0.3

### Microbiological findings

A causative pathogen was identified in 274 episodes (94.2%), whereas 17 (5.8%) were culture-negative. Among culture-positive cases, 51.1% were Gram-positive, 40.5% Gram-negative, and 8.4% fungal (all *Candida* spp.) (Table 2).

**Table 2:** Microbiological profile of pediatric catheter-related infection episodes

Variable	*n*	%
Total CRI episodes	291	100
Culture result		
Positive	274	94.2
Negative	17	5.8
Distribution of pathogens (culture-positive episodes, ***n*** = 274)		
Gram-positive bacteria	140	51.1
Coagulase-negative staphylococci	89	32.5
*Staphylococcus aureus*	11	4.0
*Enterococcus* spp.	25	9.1
Other Gram-positive bacteria	15	5.5
Gram-negative bacteria	111	40.5
Enteric Gram-negative bacteria	58	21.1
*Pseudomonas* spp.	15	5.5
*Acinetobacter* spp.	14	5.0
Other Gram-negative bacteria	24	8.8
Fungal pathogens (***Candida*** spp.)	23	8.4

Antimicrobial resistance was assessed for clinically relevant pathogen–antibiotic pairs, including methicillin resistance in *staphylococci*, vancomycin resistance in *enterococci*, and extended-spectrum β-lactam and carbapenem resistance among Gram-negative organisms. Resistance rates were calculated as the proportion of resistant isolates among those tested for each antimicrobial agent, based on EUCAST-defined breakpoints. Overall, antimicrobial resistance was detected in a subset of isolates, including methicillin-resistant *Staphylococcus aureus* (0.7%), methicillin-resistant coagulase-negative staphylococci (1.0%), vancomycin-resistant *Enterococcus* spp. (3.7%), extended-spectrum β-lactamase–producing *Escherichia coli* (3.0%), and carbapenemase-producing *Klebsiella pneumoniae* (1.3%).

### Associations with pathogen class

Antibiotic exposure at catheter insertion was less frequent in Gram-positive CRI (36.4%) than in Gram-negative (52.3%) and fungal infections (56.5%; *p* = 0.021). Immunosuppression was more common in bacterial CRI (Gram-positive 72.1%; Gram-negative 73.9%) than in fungal CRI (39.1%; *p* = 0.003).

Permanent catheters predominated in bacterial CRI (Gram-positive 81.4%; Gram-negative 74.8%), whereas fungal CRI was more frequently associated with temporary lines (52.2%; *p* = 0.002). Triple-lumen catheters were disproportionately represented in fungal CRI (52.2%; *p* = 0.001). Clinical and device-related correlates are presented in Table 3.

**Table 3:** Impact of clinical and catheter characteristics on the causative pathogen in catheter‑related infection

Variable, *n* (%)	Gram-positive (*n* = 140)	Gram-negative (*n* = 111)	Fungal (*n* = 23)	*p* value
Altered consciousness	3 (2.1)	9 (8.1)	3 (13.0)	0.020
Respiratory failure	5 (3.6)	9 (8.1)	2 (8.7)	0.020
Immunosuppression	101 (72.1)	82 (73.9)	9 (39.1)	0.003
Antibiotic exposure at insertion	51 (36.4)	58 (52.3)	13 (56.5)	0.021
Catheter type				0.002
Permanent	114 (81.4)	83 (74.8)	11 (47.8)	
Temporary	26 (18.6)	28 (25.2)	12 (52.2)	
Lumen count				0.001
2 lumens	122 (87.1)	91 (82.0)	11 (47.8)	
3 lumens	18 (12.9)	20 (18.0)	12 (52.2)	

### Laboratory findings at infection onset

Absolute leukocyte, neutrophil, monocyte, platelet counts, and erythrocyte sedimentation rate did not differ significantly across pathogen groups. Lymphocyte counts were highest in fungal CRI (*p* = 0.02). NLR demonstrated a markedly right-skewed distribution in Gram-negative CRI (median 3; range 0–10410). CRP and PCT levels were significantly elevated in Gram-negative CRI compared with Gram-positive and fungal CRI (*p* = 0.03 and *p* = 0.001, respectively). Laboratory parameters are summarized in Table 4.

**Table 4:** Inflammatory biomarkers and their discriminatory performance for pathogen class

Comparison	Parameter	AUC (95% CI)	*p* value	Cut-off	Sensitivity	Specificity
Gram-positive vs. fungal	Lymphocyte	0.676 (0.561–0.791)	0.013	705	0.84	0.34
	NLR	0.567 (0.456–0.677)	0.348	1.26	0.84	0.45
	CRP	0.638 (0.505–0.771)	0.052	74.11	0.52	0.76
	PCT	0.551 (0.411–0.691)	0.478	0.79	0.52	0.65
	Time from catheter insertion to infection	0.317 (0.198–0.435)	0.010	24.5	0.31	0.34
Gram-negative vs. fungal	Lymphocyte	0.738 (0.622–0.855)	0.001	725	0.84	0.606
	NLR	0.452 (0.342–0.561)	0.505	0.711	0.89	0.32
	CRP	0.508 (0.375–0.640)	0.915	23.44	0.79	0.33
	PCT	0.380 (0.239–0.522)	0.099	49.69	0.16	0.88
	Time from catheter insertion to infection	0.354 (0.228–0.480)	0.044	23.5	0.42	0.36
Gram-negative vs. Gram-positive	Lymphocyte	0.468 (0.393–0.544)	0.416	15	0.85	0.22
	NLR	0.583 (0.507–0.659)	0.032	2.456	0.58	0.59
	CRP	0.618 (0.544–0.692)	0.002	78.7	0.43	0.78
	PCT	0.663 (0.593–0.734)	0.001	1.56	0.58	0.71
	Time from catheter insertion to infection	0.453 (0.377–0.528)	0.221	1.5	0.99	0.02

*AUC*, area under the receiver operating characteristic curve; *CI*, confidence interval; *NLR*, neutrophil-to-lymphocyte ratio; *CRP*, C-reactive protein; *PCT*, procalcitonin

### Multivariable analysis

The Gram-negative versus Gram-positive model included culture-positive bacterial episodes only, whereas the fungal versus bacterial model included all culture-positive cases.

In adjusted analyses, CRP (AOR 1.005, 95% CI 1.001–1.009; *p* = 0.017) and NLR (AOR 1.037, 95% CI 1.006–1.069; *p* = 0.019) were independently associated with Gram-negative infection. PCT did not retain significance after adjustment (AOR 1.007, 95% CI 0.995–1.020; *p* = 0.258).

### Discriminatory performance

ROC analysis demonstrated modest discrimination between Gram-negative and Gram-positive CRI. PCT showed the highest performance (AUC 0.663, 95% CI 0.593–0.734; *p* = 0.001), followed by CRP (AUC 0.618, 95% CI 0.544–0.692; *p* = 0.002) and NLR (AUC 0.583, 95% CI 0.507–0.659; *p* = 0.032). Absolute lymphocyte count and catheter duration did not demonstrate significant discriminatory capacity. For fungal versus bacterial CRI, lymphocyte count demonstrated moderate discrimination (AUC 0.711; *p* = 0.002), whereas CRP, NLR, and PCT showed limited performance.

Using Youden-derived cut-offs (PCT 1.56 ng/mL; CRP 78.7 mg/L; NLR 2.456), elevated PCT was associated with increased odds of Gram-negative infection (AOR 2.79, 95% CI 1.506–5.183; *p* = 0.001), whereas dichotomized CRP and NLR did not retain significance.

### Clinical outcomes

Overall, 56% of episodes achieved remission, 14.4% relapsed, and 5.8% were associated with 30-day mortality. Catheter removal occurred in 26.1% of episodes and was universal in fungal CRI (*p* < 0.001). Remission rates differed across pathogen groups (*p* < 0.01), and relapse was more frequent in Gram-negative than in Gram-positive CRI (*p* < 0.01).

## Discussion

Catheter-related infections in pediatric populations pose a substantial therapeutic challenge because empirical antimicrobial therapy must often be initiated before microbiological confirmation. In the absence of pathogen-stratified clinical frameworks, early decisions rely largely on nonspecific indicators. This study aimed to delineate organism-class–specific clinical, device-related, and inflammatory phenotypes to improve early risk stratification.

The observed incidence density of 11.7 per 1000 catheter days reflects the high-risk tertiary case mix of our institution and provides epidemiological context for the pathogen-specific phenotypes described. Our findings indicate that pediatric CRI exhibit distinct pathogen-associated patterns. Gram-negative infections were characterized by a more pronounced systemic inflammatory response, reflected by elevated CRP, PCT, and NLR levels [25].

In contrast, fungal CRI demonstrated a distinct immuno-inflammatory and device-related profile, with higher lymphocyte counts and a disproportionate association with temporary and triple-lumen catheters, as well as universal early catheter removal. These findings support the concept that CRI phenotypes reflect interactions between pathogen biology, host immunity, and device characteristics.

Sex was not associated with pathogen distribution in our cohort. Although Cabrero et al. reported male sex as a potential risk factor in one study within a large meta-analysis, overall evidence remains inconsistent [15], and our findings suggest that sex is unlikely to be a major determinant of pathogen class in pediatric CRI.

The high prevalence of hematologic, oncologic, and immunologic disorders reflects the tertiary referral nature of our institution. Compared with other Turkish cohorts [24, 26], our population included a higher proportion of children with complex underlying diseases, likely influencing catheter utilization and pathogen distribution. For example, Mısırlıoğlu et al. reported that 38% of catheter insertions occurred in hematology-oncology-immunology wards [27], substantially lower than in our setting. These differences underscore the importance of institutional case mix when interpreting CRI epidemiology.

Insertion-related variables such as TPN, prior surgery, and skin integrity disruption were not associated with pathogen class, despite established links between TPN and central venous CRI in guideline statements [28, 29]. The relatively low TPN prevalence in our cohort may have limited statistical power. In contrast, antibiotic exposure at catheter insertion was associated with a relative shift toward Gram-negative and fungal CRI, consistent with antimicrobial ecological pressure. Baier et al. demonstrated a sixfold increase in CVC-BSI risk with peri-insertional carbapenem use [16], supporting the hypothesis that early antibiotic exposure may influence pathogen selection.

Immunosuppression was more frequent in bacterial than fungal CRI (*p* = 0.003), aligning with prior reports linking immune dysfunction and stem-cell transplantation to increased CRI risk [30, 31]. However, the specific cause of immunosuppression did not independently determine pathogen class, suggesting that immune compromise enhances infection susceptibility without dictating microbiological phenotype.

Fungal CRI exhibited a distinct clinical and device-related profile. Invasive candidiasis is well recognized to be associated with central venous access, intensive care exposure, TPN, and broad-spectrum antibiotic use [32]. In our cohort, fungal infections were disproportionately linked to temporary and multi-lumen devices, and catheter removal was uniformly required, consistent with candidemia management recommendations. Despite this severity profile, no 30-day mortality was observed, possibly reflecting prompt device removal and antifungal therapy.

Although inflammatory biomarkers differed across pathogen groups, their discriminatory performance was modest. ROC analyses demonstrated substantial overlap in biomarker distributions. While PCT achieved the highest univariable AUC, it lost independent significance after multivariable adjustment, likely due to collinearity with CRP and NLR. The markedly right-skewed distribution of NLR in Gram-negative CRI further indicates potential outlier influence and warrants cautious interpretation.

Most pediatric CRI studies compare infected and non-infected catheter populations; few focus on pathogen-class discrimination within confirmed CRI. By restricting analysis to microbiologically confirmed episodes, our findings emphasize the limited standalone predictive value of individual biomarkers. Integrated multivariable approaches combining host, device, and inflammatory variables may provide a more clinically meaningful framework for contextual risk assessment during initial management.

Outcome analyses also revealed pathogen-specific differences. Christison-Lagay et al. reported catheter salvage rates of 60–69% with antibiotic lock therapy in oncology populations [33]. In contrast, fungal CRI in our cohort required near-universal catheter removal and exhibited lower remission rates. Relapse was more frequent in Gram-negative CRI, potentially reflecting differences in virulence, biofilm formation, and antimicrobial penetration. Prior studies have similarly reported increased relapse risk in coagulase-negative staphylococcal CRI without significant mortality impact [11, 34]. These data support selective catheter preservation in certain bacterial infections but reinforce the limited role of salvage in fungal disease. The higher relapse rate in Gram-negative CRI may relate to enhanced biofilm dynamics and antimicrobial resistance patterns. Although neutropenia did not independently predict fungal infection, impaired innate immunity remains a recognized contributor to invasive fungal disease.

Several limitations should be acknowledged. The episode-based design introduces potential within-patient correlation, as 36.7% of patients experienced more than one CRI episode. Although each episode was analyzed independently to preserve statistical power, the absence of clustered or mixed-effects modeling may have resulted in underestimated standard errors. Therefore, effect size estimates should be interpreted cautiously. Resistance patterns were described but not incorporated into predictive models. Calibration metrics and validation procedures were not performed, raising the possibility of model optimism. Finally, moderate AUC values underscore the limited standalone discriminatory capacity of individual biomarkers.

Strengths of this study include detailed episode-level phenotyping, comprehensive catheter characterization, and pathogen-stratified multivariable modeling within a large tertiary pediatric cohort. Integration of inflammatory markers with device-related variables may enhance contextual risk assessment in suspected CRI.

From a pragmatic perspective, markedly elevated CRP and PCT levels accompanied by high NLR may increase suspicion for Gram-negative CRI, whereas temporary multi-lumen catheter use may heighten concern for fungal infection. However, these features should be interpreted within a structured multivariable framework rather than in isolation.

In conclusion, pediatric CRI demonstrate biologically plausible pathogen-specific inflammatory and device-related phenotypes. Nonetheless, biomarker discrimination remains modest, and overlapping distributions limit standalone predictive utility. Integrated profiling approaches may inform contextual assessment at first suspicion but require prospective multicenter validation with formal calibration before clinical implementation.

## Data Availability

Detailed data are available upon request from the corresponding author.
